# Subcellular Expression Patterns of FKBP Prolyl Isomerase 10 (FKBP10) in Colorectal Cancer and Its Clinical Significance

**DOI:** 10.3390/ijms241411415

**Published:** 2023-07-13

**Authors:** Yating Fu, Jiahui Chen, Xianhua Ma, Wenjun Chang, Xiongbao Zhang, Yu Liu, Hao Shen, Xuefei Hu, An-Jing Ren

**Affiliations:** 1 Department of Navy Environmental and Occupational Health, Faculty of Naval Medicine, Naval Medical University, Shanghai 200433, China; fyt@smmu.edu.cn (Y.F.); 15647463109@163.com (J.C.); cwjcwj1976@smmu.edu.cn (W.C.); xiongbao20202020@163.com (X.Z.); liuyu_724@163.com (Y.L.); shshao1990@163.com (H.S.); 2 Department of Pathophysiology, College of Basic Medical Sciences, Naval Medical University, Shanghai 200433, China; maxhuacn@aliyun.com; 3 Experimental Teaching Center, College of Basic Medical Sciences, Naval Medical University, Shanghai 200433, China

**Keywords:** colorectal cancer, the FK506-binding proteins (FKBPs), FKBP10, prognosis, subcellular expression pattern

## Abstract

FKBP10, a member of the FK506-binding protein (FKBP) family, has been implicated in cancer development, although its prognostic function remains controversial. In this study, we analyzed the expression of FKBP10 in tumor tissues using online databases (TCGA) as well as our CRC cohort, and investigated the relationship between its subcellular expression pattern and patient outcomes. Cox regression analysis was used to determine the associations between different subcellular expression patterns of FKBP10 and clinical features of patients. We also discussed the expression level of FKBP10 based on different subcellular expression patterns. Our results showed that FKBP10 was significantly elevated in CRC tissues and exhibited three different subcellular expression patterns which were defined as ‘FKBP10-C’ (concentrated), ‘FKBP10-T’ (transitional) and ‘FKBP10-D’ (dispersive). The FKBP10-D expression pattern was only found in tumor tissues and was associated with unfavorable disease-free survival in CRC patients. High expression levels of FKBP10-C predicted an unfavorable prognosis of recurrence of CRC, while FKBP10-D did not. Our findings suggest that the subcellular expression patterns and expression level of FKBP10 play crucial prognostic roles in CRC, which revealed that FKBP10 may be a viable prognostic and therapeutic target for the diagnosis and treatment of CRC.

## 1. Introduction

In recent years, colorectal cancer (CRC) has emerged as the second leading cause of cancer-related deaths and is projected to become the third most common adult cancer in America in 2023 [1]. Despite significant advancements in endoscopy, surgery, preoperative radiotherapy and neoadjuvant chemotherapy, the pathophysiology of CRC remains a subject of intense research [2]. The identification of pathophysiological biomarkers such as EGFR (Cetuximab), VEGFR (Bevacizumab) and SHP2 (TNO155, NCT04330664, etc., phase II clinical trials) has expanded the range of individual treatment options. However, the search for novel therapeutic biomarkers continues, as it promises to provide new insights into the diagnosis and treatment of CRC.

One such biomarker is FKBP10, a member of the FK506-binding protein (FKBP) family that binds immunosuppressive drugs such as FK506 and rapamycin, and exhibits peptidyl prolyl isomerase (PPIase) activity [3]. FKBPs are chaperone molecules that participate in various cellular process such as protein folding, protein stability, cellular signaling, apoptosis and transcription, and thus contribute to diverse diseases including inflammation, fibrosis development, neurodegenerative disorders and cancer [3,4,5,6]. Increasing evidence suggests that FKBPs are associated with numerous human malignancies. For example, the transcriptional levels of FKBP7 [7], FKBP11 [8] and FKBP15 [9] are elevated in prostate cancer, renal cell cancer and breast cancer, respectively. FKBP5 is a prognostic indicator in pancreatic cancer and breast cancer, while FKBP9 is linked to poor prognoses in prostate cancer and gliomas [10,11,12]. Wang et al. utilized high-throughput bioinformatics technology to investigate the prognostic correlations of FKBP genes in lung adenocarcinoma and found that FKBP2, FKBP3, FKBP4, FKBP10, FKBP11 and FKBP14 were overexpressed in lung cancer, with FKBP10 showing a correlation with poor patient prognosis [4]. FKBP10, an endoplasmic reticulum (ER) chaperone protein containing four PPIase domains, has been shown to be closely associated with cancer development. FKBP10 is overexpressed in renal cancer [8,13], glioma [14] and gastric cancer [15,16], where it promotes tumor cell proliferation, invasion and migration. In lung cancer, FKBP10 is selectively expressed and negatively correlated with patients’ outcomes [17]. In CRC, FKBP10 is one of the prognostic markers for overall survival and comprises the signatures for CRC prognosis prediction [18,19]. Chen et al. also found that FKBP10 was highly expressed in CRC tissues and associated with poor prognosis [20]. However, the function of FKBP10 in cancer is still under debate. Quinn et al. reported that FKBP10 was downregulated in ovarian cancer and suggested that the low expression level of FKBP10 might be associated with tumorigenicity [21]. Therefore, more research is required to determine the significance of FKBP10 in cancer. In this study, we evaluated the association between FKBP10 expression, clinical features and patient survival in a CRC cohort (n = 682), taking into consideration not only the abundance of FKBP10 but also its subcellular expression patterns. The global intracellular FKBP10 was increased in colon adenoma and CRC tissues compared with normal tissues. Further investigations showed that there were three distinct expression patterns of FKBP10, which were defined as ‘FKBP10-C’ (concentrated), ‘FKBP10-T’ (transitional) and ‘FKBP10-D’ (dispersive). The expression of FKBP10-D was found to be significantly higher in CRC tissues, and was associated with poor patient outcomes. Additionally, the upregulation of FKBP10-C was also indicative of a poor prognosis in the subcohort. These findings suggest that FKBP10 plays a crucial role in CRC prognosis and may be a promising therapeutic target for the diagnosis and treatment of CRC.

## 2. Results

### 2.1. FKBP10 Exhibited Different Subcellular Expression Patterns in CRC Tissues

**Figure 1:**
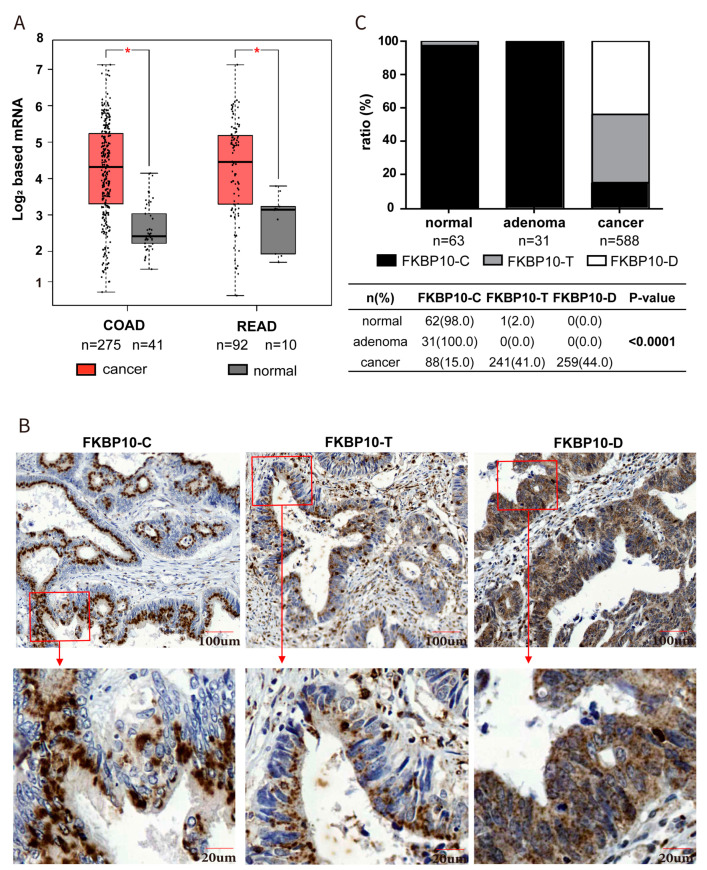
FKBP10 was elevated in CRC tissues and exhibited three different expression patterns. (**A**) FKBP10 mRNA level in colon cancer and rectal cancer based on TCGA-CRC cohort. (**B**) Representative images of immunostaining of the concentrated (FKPB10-C), transitional (FKBP10-T) and dispersive FKBP10 (FKPB10-D) of CRC tissue in our cohort. (**C**) The proportion of FKPB10-C, FKPB10-T and FKPB10-D in normal, adenoma and tumor tissues. * *p* < 0.05.

Firstly, we analyzed the transcriptional expression of FKBP10 in normal and CRC tissues based on a TCGA-CRC dataset (n = 418, GEPIA). The results showed that the global transcriptional expression of FKBP10 was significantly elevated in colon adenocarcinoma (COAD) and rectum adenocarcinoma (READ) tissues compared with normal tissues, indicating a tumor promotive function of FKBP10 (Figure 1A). We then detected the translational expression of FKBP10 in our CRC cohort (n = 682) through IHC staining. As shown in Figure 1B, FKBP10 was mainly expressed in epithelial cells in colonic and rectal tissues, with the epithelial FKBP10 exhibiting different subcellular expression patterns. In some tissues, FKBP10 was ‘concentrated’ (FKBP10-C), presenting as round-, oval- or spindle-shaped and arranged neatly near the nucleus. However, in some tissues, FKBP10 was ‘dispersive’ (FKBP10-D), distributed diffusely in the cytoplasm. In the other tissues, FKBP10 was ‘transitional’ (FKBP10-T), and exhibited being ‘concentrated’ and ‘dispersive’ at the same time. We then compared the subcellular expression patterns of FKBP10 in normal, adenoma and CRC tissues and found that in almost all normal (98.4%) and adenoma (100%) tissues, FKBP10 was expressed as ‘FKBP10-C’, while only 15% of CRC tissues exhibited ‘FKBP10-C’ expression. Meanwhile, FKBP10-D and FKBP10-T were only observed in tumor tissues, with percentages of 44% and 41%, respectively (Figure 1C, *p* < 0.0001). These results suggest that the epithelial FKBP10 only exhibits three subcellular expression patterns in tumor tissues, which may contribute to the prognosis of CRC.

### 2.2. Subcellular Expression Patterns of FKBP10 Were Correlated with Differentiation Grade, TNM Stage and Serum Tumor Markers of CRC Patients

We further analyzed the associations between subcellular expression patterns of FKBP10 and clinicopathological characteristics of CRC patients using the Pearson χ^2^ test (for categorical variables) or Mann–Whitney U test (for non-parametric variables). As shown in Table 1, the FKBP10-D expression was significantly associated with a poor differentiational grade (*p* < 0.001), and there was a gradual increase in the percentage of patients with TNM stage Ⅲ in the FKBP10-C, FKBP10-T and FKBP10-D groups (*p* < 0.001). Additionally, FKBP10-D expression was associated with higher serum CEA and CA19-9 levels (both *p* < 0.05), but no differences were observed in terms of age, gender, chemotherapy or cancer location. The findings indicate a promotive function of FKBP10-D in CRC development.

### 2.3. Dispersive Expression of FKBP10 Was an Independent Risk Factor Which Predicted Unfavorable Disease-Free Survival in CRC

**Figure 2:**
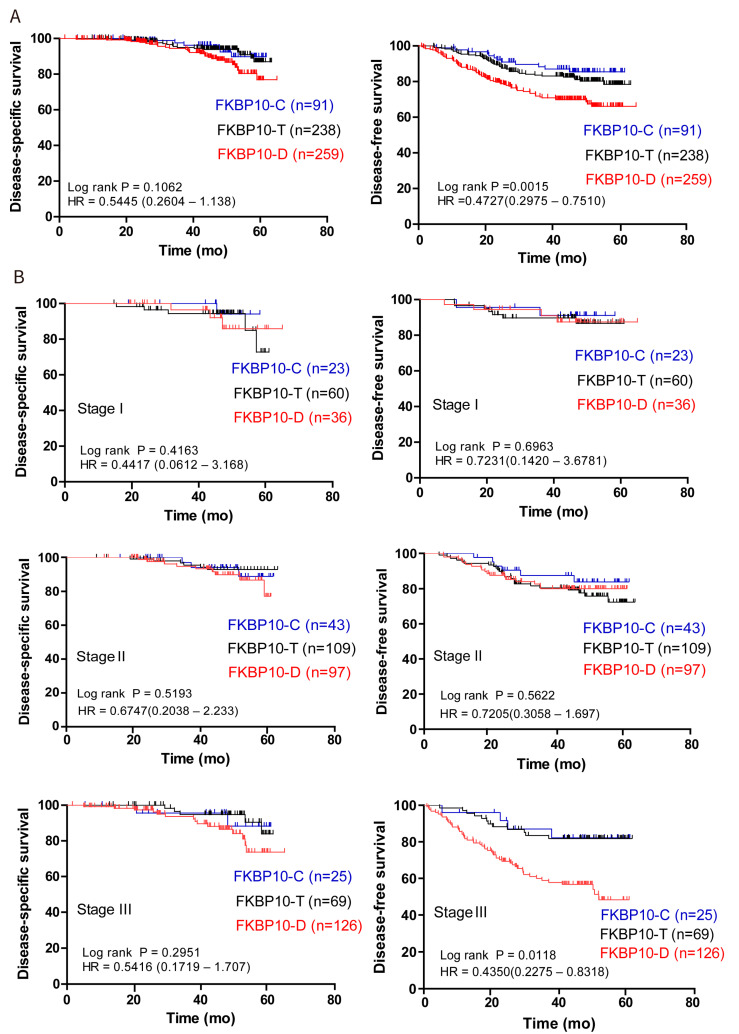
FKBP10-D expression pattern indicated unfavorable prognosis in CRC. (**A**) The associations of FKBP10 subcellular expression patterns with the overall DSS and DFS of CRC patients. (**B**) The associations of FKBP10 subcellular expression patterns with DSS and DFS in TNM stage Ⅰ, Ⅱ and Ⅲ of CRC patients. Log-rank P values were representative of three subgroups and hazard ratios (HRs) were representative of FKPB10-C vs. FKPB10-D from Kaplan–Meier analysis with log-rank test.

Next, we investigated the correlation between subcellular expression patterns of FKBP10 and the clinical outcome of CRC patients. Kaplan–Meier analysis was utilized to demonstrate the impact of different FKBP10 expression patterns on DSS and DFS. As shown in Figure 2A, patients with FKBP10-D expression exhibited slightly worse DSS but significantly shortened DFS. Successively, we investigated the prognostic value of FKBP10 expression patterns in relation to different TNM stages, tumor location and the adoption of chemotherapy. The DFS of patients who expressed FKBP10-D showed enlarged differences compared with patients who expressed FKBP10-C and FKBP10-T in TNM stage Ⅲ, although no contribution of FKBP10-D to DFS was found in stage I and II (Figure 2B). In addition, FKBP10-D expression remarkably contributed to the DFS of rectal cancer and patients who received chemotherapy (Figure S1). Meanwhile, we carried out univariate and multivariate Cox regression analysis to explore the associations between subcellular expression patterns of FKBP10, clinical features and the survival of patients. The subcellular expression patterns of FKBP10 (*p* < 0.001), along with the TNM stage (*p* < 0.001), differentiation grade (*p* < 0.001), chemotherapy (*p* < 0.05), tumor location (*p* < 0.05), serum CEA (*p* < 0.001) and serum CA19-9 (*p* < 0.001) were significantly associated with the DFS of patients via univariate Cox analysis. Multivariate Cox analyses showed that, except for chemotherapy, tumor location and serum CEA, the other four features were independent risk factors in CRC. Moreover, the FKBP10 expression patterns (*p* < 0.05), serum CEA (*p* < 0.05) and serum CA19-9 (*p* < 0.05) were risk factors for unfavorable DSS of patients. Importantly, compared with the FKBP10-C plus FKBP10-T group, the FKBP10-D expression pattern predicted poorer survival with an HR of 1.794 (95% CI, 1.012–3.180, *p* < 0.05) to DSS and an HR of 1.599 (95%CI, 1.111–2.200, *p* < 0.05) to DFS in CRC (Table 2).

### 2.4. Elevation of Concentrated FKBP10 Indicated Poor Prognosis in CRC

**Figure 3:**
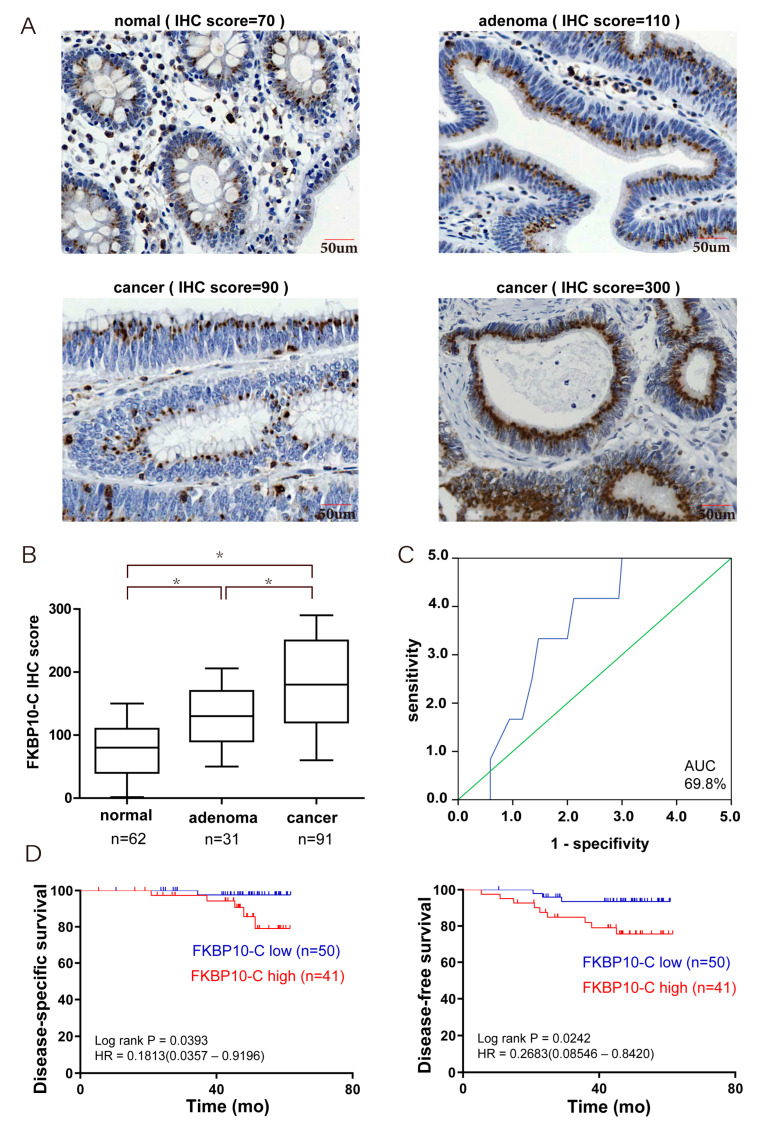
Highly expressed epithelial FKBP10-C predicted unfavorable prognosis in CRC. (**A**) Representative images of immunostaining of the FKBP10-C in normal, adenoma and cancer tissues, respectively. (**B**) IHC scores of epithelial FKBP10-C expression in normal, adenoma and cancer tissue in the CRC cohort; (**C**) ROC curve analysis of epithelial FKBP10-C expression and patients’ outcome in the CRC cohort. green line: reference line; blue line: samples’ ROC curve. (**D**) The associations of epithelial FKBP10-C expression with the overall DSS and DFS of CRC patients. * *p* < 0.05.

Although the subcellular expression pattern of FKBP10 indicated poor prognosis in CRC, the contribution of the expression level of FKBP10 to CRC remains unclear in our cohort. Therefore, we further evaluated the expression level of FKBP10-C and FKBP10-D to explore their influence on DFS or DSS in patients, respectively. The typical IHC staining of normal, adenoma and cancer tissue with low or high FKBP10-C is shown in Figure 3A, respectively. Among the various tissue types of the CRC cohort, the level of FKBP10-C was the lowest in normal tissue and significantly increased in adenoma and cancer tissues (Figure 3B). Based on the 69.8% AUC of the ROC curve, the FKBP10-C specimens were classified into subgroups of high FKBP10-C (IHC score ≥ 200, n = 41) or low FKBP10-C (IHC score < 200, n = 50), and the high FKBP10-C subgroup showed significantly poorer DFS and DSS than the low FKBP10-C subgroup (Figure 3C,D). Since the FKBP10-D expression pattern only existed in tumorous tissues, representative pictures of different IHC staining of cancer patients are shown in Figure S2A. ROC curve analysis showed an AUC of 50.9% and the high FKBP10-D subgroup (IHC score ≥ 155, n = 70) exhibited no prognostic difference with the low FKBP10-D subgroup (IHC score < 155, n = 189) in terms of DFS and DSS (Figure S2B,C). In summary, the elevation of the concentrated expression of FKBP10 indicated poor prognosis in CRC, while the dispersive expression of FKBP10 did not.

## 3. Discussion

Several decades ago, due to the insidious development and simple metastasis of CRC, patients were usually diagnosed at an advanced stage. The widespread use of endoscopic, extensive surgical, downstaging preoperative radiotherapy and systemic therapy has improved the overall survival for advanced disease to 3 years. However, survival is still best for those with non-metastasized disease [2]. Enormous efforts have been made to identify novel therapeutic targets for the diagnosis and treatment of CRC.

FKBPs are a protein family that possess the protein folding property of peptidyl-prolyl cis-trans isomerization. FKBP12, the prototype of this family, is considered to be the minimum domain structure of FKBPs. The single FK506-binding domain (FKBD) of FKBP12 binds drugs like FK506 and rapamycin, acting as an immunosuppressive effect [3]. Elevating evidence suggests that FKBPs are also risk factors and biomarkers for cancer, including CRC. FKBPs play crucial roles in biological processes and carcinogenesis; however, their prognostic value and molecular mechanism in cancer are still poorly understood. FKBP10 is one of the FKBPs that contains four PPIase domains. FKBP10 mutation causes collagen-related illnesses such as osteogenesis imperfecta by decreasing collagen secretion [22]. FKBP10 is a resident protein of the ER lumen and acts as a molecular chaperone [6]. The ER is the major site of protein synthesis, folding and assembly. Disorganization of the ER occurs in many pathologies associated with an obstruction of the ER-to-Golgi or intra-Golgi transport, including neurological disorders and cancer [23]. Along with the association with ribosomes and regulation of translation elongation, particularly upon the insertion of proline, FKBP10 has been proven to promote cancer growth and stemness via its PPIase activity [17]. FKBP10 has also been reported to be associated with cancer development, especially in gastrointestinal cancers. More abundant FKBP10 has been detected and has revealed correlations with poor prognosis in gastric cancer, stomach adenocarcinoma and CRC [15,16,19,20]. 

Considering the function of FKBP10, we analyzed the subcellular expression patterns of this protein. We found that FKBP10 (FKBP10-C) was concentrated near the nucleus in most colorectal tissues. As an ER resident protein, the distribution of FKBP10 in cancer tissues was consistent with its role. However, a dispersive expression of FKBP10 (FKBP10-D) was observed in 44% of cancer tissues, indicating a change in function and potential impact on the development of CRC. Thus, we explored the association between subcellular expression patterns of FKBP10 and the prognosis of patients in our CRC cohort. The results showed that the expression pattern of FKBP10 was an independent risk factor in CRC, with FKBP10-D predicting an unfavorable prognosis of recurrence. The different subcellular expression patterns of FKBP10 in the cytoplasm may reveal physiological or pathological changes in the ER, such as ER stress, which is closely related to the occurrence and development of CRC [24]. 

Furthermore, we analyzed the significance of FKBP10-C and FKBP10-D in CRC separately. In the FKBP10-C subgroup, the overexpression of FKBP10-C was detected in cancer tissues compared to normal tissues and indicated an unfavorable prognosis in CRC. However, in the FKBP10-D subgroup, the expression level of FKBP10 was not prognostic in CRC. These results suggest that the expression patterns of FKBP10 vary substantially between cancer and normal tissues and are prognostic in CRC, with the dispersive expression of FKBP10 exhibiting a link with unfavorable survival. Additionally, for those patients who express concentrated FKBP10, the expression level of this protein could also be a predictor of their outcome, with higher FKBP10-C indicating poorer survival. 

## 4. Materials and Methods

### 4.1. Bioinformatics

The processed data were obtained from TCGA data available from the website of Gene Expression Profiling Interactive Analysis (GEPIA). The row data were provided for further analysis from TCGA-COAD (colon adenocarcinoma), containing 275 tumor tissues and 41 normal tissues, and TCGA-READ (rectum adenocarcinoma), containing 92 tumor tissues and 10 normal tissues.

### 4.2. Patients and Specimens

A total of 682 patients who were diagnosed at Changhai Hospital between January 2006 and November 2011 were included in the study. Formalin-fixed, paraffin-embedded (FFPE) tissues were obtained from 63 normal, 31 adenoma and 588 cancerous samples. Patients who received preoperative chemotherapy or radiation were excluded from the study. The patients’ characteristics, including age, gender, differentiational grade, TNM stage (according to the American Joint Committee on Cancer Staging System, 7th edition), adjuvant chemotherapy, tumor location, serum carcinoembryonic antigen (CEA) and carbohydrate antigen 19-9 (CA19-9) levels, were collected. The follow-up information of the 588 CRC patients was also collected in the same manner as described in a previous study [25]. Disease-free survival (DFS) was used as the relative outcome of recurrence, defined as the duration (in months) from the completion of surgery to the date of the first relapse. Disease-specific survival (DSS) was used as the relative outcome for death, defined as the duration (in months) from the completion of surgery to the date when the patient died of CRC. The study was approved by the Institutional Review Committee of Changhai Hospital, and all participants signed a written informed consent form allowing the use of biomaterials in the study. 

### 4.3. Immunohistochemistry

Based on the FFPE specimens, tissue microarrays (TMAs) were constructed using a commercial agent (Outdo Biotech, Shanghai, China) for immunohistochemistry (IHC) examination of FKBP10 expression. Briefly, slides with standard 4 µm thick sections were first baked at 85 °C for 30 min, deparaffinized using xylene and rehydrated in a graded ethanol series. After blocking endogenous peroxidase for 5 min with 3% H_2_O_2_, the FKBP10 antigen was retrieved by boiling all slides in sodium citrate (10 mmol/L, pH 6.0, 100 °C) for 30 min. The slides were then incubated overnight at 4 °C with rabbit anti-human FKBP10 polyclonal antibody (1:1000, ab230852; Abcam, Cambridge, UK) according to the manufacturer’s instructions. The antibody specificity data can be found on The Human Protein Atlas website. After incubation with secondary antibodies from the ElivisionTMsuper HRP (Mouse/Rabbit) IHC Kit (kit-9922; Maxvision, Foshan, China) for 30 min, the slides were reacted in diaminobenzidine (DAB) solution for 45 s and stained with hematoxylin for 25 s. To eliminate intra-assay variation, all slides were stained simultaneously by the same investigator.

### 4.4. Quantitative Evaluation of FKBP10 Immunostaining

The stained TMA slides were scanned using the Servicebio system, and ImageScope software v12.4.0.5043 was used to evaluate the images under bright-field microscopy at a resolution of ×200. The FKBP10 protein was quantified using the H-score method as in the previous study [19]. Briefly, the H-score (range: 0–300) was calculated by multiplying the average percentage of positive epithelial cells by the corresponding staining intensity—negative (0), weakly positive (1+), moderately positive (2+) and strongly positive (3+). Two independent investigators, who were blinded to the clinical follow-up information, evaluated all slides simultaneously, and the difference between the observers was averaged.

### 4.5. Statistical Analysis

The independent sample t-test (for two groups) and the analysis of variance (for three groups) were used to compare continuous variables, such as H-score, among CRC samples. The paired t-test was used for paired samples between cancer and normal tissues. Categorical variables, such as gender and tumor differentiation grade, were compared and analyzed using the chi-square or Mann–Whitney U test. For survival analysis, the ROC curve was used to determine the optimal cut-off value of the IHC score to identify the risk subgroups. The Kaplan–Meier curves were utilized to assess the statistical significance of the survival curves using the log-rank test. The hazard ratios (HRs) and corresponding 95% confidence intervals (CIs) were estimated using Cox proportional hazards models, both univariate and multivariate. All statistical tests were two-sided and performed using IBM SPSS statistics 23 for Windows. Statistical significance was set at *p* < 0.05.

## 5. Conclusions

In this study, we systematically described the expression patterns and clinical significance of FKBP10 in CRC and creatively found that the expression of FKBP10 in CRC tissue exhibits various distribution patterns: concentrated (FKBP10-C), transitional (FKBP10-T) and dispersive (FKBP10-D), which were related to the intracellular localization and function of FKBP10 in epithelium. Our results showed that the FKBP10-D expression pattern, as well as the overexpression of FKBP10-C, indicated an unfavorable prognosis in our CRC cohort. This suggests that not only the expression level but also the subcellular expression patterns of FKBP10 are risk factors for CRC. Further studies are needed to understand the underlying mechanisms. For the identification of and therapy for CRC, FKBP10 may be an appealing prognostic and therapeutic target.

## Figures and Tables

**Table 1:** Characteristics of patients separated by the subcellular expression patterns of FKBP10 in CRC cohort.

Variables	All (n = 588)			
	FKBP10-C	FKBP10-T	FKBP10-D	*p*-Value
	(n = 91)	(n = 238)	(n = 259)	
Age, mean ± SD	60.31 ± 11.67	60.21 ± 12.38	61.62 ± 12.55	0.398
Sex, n (%)				0.256
Male	59 (64.8)	137 (57.6)	166 (64.1)	
Female	32 (35.2)	101 (42.4)	93 (35.9)	
Differential grade, n (%)				<0.0001
Well	2 (2.2)	2 (0.8)	0 (0.0)	
Moderate	89 (97.8)	234 (98.3)	244 (94.2)	
Poor	0 (0.0)	2 (0.8)	15 (5.8)	
TNM stage, n (%)				<0.0001
I	23 (25.3)	60 (25.2)	36 (13.9)	
II	43 (43.7)	109 (45.8)	97 (37.5)	
III	25 (27.5)	69 (29.0)	126 (48.6)	
Chemotherapy, n (%)				0.077
Yes	56 (61.5)	157 (66.0)	189 (73.0)	
No	35 (38.5)	81 (34.0)	70 (27.0)	
Serum CEA, n (%)				0.0002
<5 ng/mL	66 (72.5)	163 (68.8)	147 (57.6)	
≥5 ng/mL	25 (27.5)	74 (31.2)	108 (42.4)	
Serum CA19-9, n (%)				0.021
<37 U/mL	79 (86.8)	216 (91.5)	212 (83.1)	
≥37 U/mL	12 (13.2)	20 (8.5)	43 (16.9)	
Location, n (%)				0.173
Rectum	66 (72.5)	147 (61.8)	163 (62.9)	
Colon	25 (27.5)	91 (38.2)	96 (37.1)	

FKPB10-C, concentrated expression of FKBP10; FKPB10-T, transitional expression of FKBP10; FKPB10-D, dispersive expression of FKBP10; CEA, serum carcinoembryonic antigen; CA19-9, carbohydrate antigen 19-9.

**Table 2:** Cox regression analysis of FKBP10 subcellular expression patterns and clinicopathological covariates in CRC cohort.

Characteristics	Disease-Specific Survival				Disease-Free Survival			
	Univariate		Multivariate		Univariate		Multivariate	
	HR (95% CI)	*p*-Value	HR (95% CI)	*p*-Value	HR (95% CI)	*p*-Value	HR (95% CI)	*p*-Value
Dispersion vs. concentration + transition	1.962 (1.117–3.447)	0.019	1.794 (1.012–3.180)	0.046	2.013 (1.421–2.852)	<0.0001	1.599 (1.111–2.200)	0.011
Age (>60 vs. ≤60 years)	0.823 (0.468–1.446)	0.497			0.925 (0.656–1.305)	0.659		
Sex (female	0.906 (0.503–1.632)	0.742			1.157 (0.816–1.641)	0.412		
vs. male)								
TNM (III vs. I + II)	1.534 (0.879–2.677)	1.132			2.156 (1.528–3.043)	<0.0001	1.686 (1.135–2.506)	0.01
Grade (poor vs. moderate + well)	1.240 (1.171–8.992)	0.831			4.913 (2.573–9.382)	<0.0001	3.451 (1.742–6.834)	<0.0001
Chemotherapy (yes vs. no)	1.199 (0.646–2.227)	0.565			1.753 (1.154–2.664)	0.006	1.050 (0.655–1.681)	0.84
Location	1.497 (0.582–2.631)	0.161			1.471 (1.038–2.086)	0.03	1.406 (0.984–2.008)	0.061
(colon vs. rectum)								
CEA (high vs. low)	1.809 (1.036–3.159)	0.037	1.555 (0.865–2.797)	0.14	1.859 (1.317–2.625)	<0.0001	1.383 (0.957–1.999)	0.084
CA19-9 (high vs. low)	2.097 (1.037–4.240)	0.039	1.553 (0.735–3.280)	0.249	2.435 (1.609–3.684)	<0.0001	1.744 (1.106–2.748)	0.017

CEA, serum carcinoembryonic antigen; CA19-9, carbohydrate antigen 19-9.

## Data Availability

The data presented in this study are available on request from the corresponding author. The data are not publicly available due to privacy restrictions.

## Supplementary Materials

The following supporting information can be downloaded at https://www.mdpi.com/article/10.3390/ijms241411415/s1.

## References

[1] Siegel R.L., Miller K.D., Wagle N.S., Jemal A. (2023). Cancer statistics 2023. CA A Cancer J. Clin..

[2] Dekker E., Tanis P.J., Vleugels J.L.A., Kasi P.M., Wallace M.B. (2019). Colorectal cancer. Lancet.

[3] Solassol J., Mange A., Maudelonde T. (2011). FKBP family proteins as promising new biomarkers for cancer. Curr. Opin. Pharmacol..

[4] Wang C., Shen W.J., Anuraga G., Hsieh Y.H., Khoa Ta H.D., Xuan D.T.M., Shen C.F., Wang C.Y., Wang W.J. (2023). Penetrating Exploration of Prognostic Correlations of the FKBP Gene Family with Lung Adenocarcinoma. J. Pers. Med..

[5] Tong M., Jiang Y. (2015). FK506-Binding Proteins and Their Diverse Functions. Curr. Mol. Pharmacol..

[6] Patterson C.E., Schaub T., Coleman E.J., Davis E.C. (2000). Developmental regulation of FKBP65. An ER-localized extracellular matrix binding-protein. Mol. Biol. Cell.

[7] Garrido M.F., Martin N.J.P., Bertrand M., Gaudin C., Commo F., El Kalaany N., Al Nakouzi N., Fazli L., Del Nery E., Camonis J. (2019). Regulation of eIF4F Translation Initiation Complex by the Peptidyl Prolyl Isomerase FKBP7 in Taxane-resistant Prostate Cancer. Clin. Cancer Res..

[8] Sun Z., Qin X., Fang J., Tang Y., Fan Y. (2021). Multi-Omics Analysis of the Expression and Prognosis for FKBP Gene Family in Renal Cancer. Front. Oncol..

[9] Ibusuki M., Fu P., Yamamoto S., Fujiwara S., Yamamoto Y., Honda Y., Iyama K., Iwase H. (2013). Establishment of a standardized gene-expression analysis system using formalin-fixed, paraffin-embedded, breast cancer specimens. Breast Cancer.

[10] Habara M., Sato Y., Goshima T., Sakurai M., Imai H., Shimizu H., Katayama Y., Hanaki S., Masaki T., Morimoto M. (2022). FKBP52 and FKBP51 differentially regulate the stability of estrogen receptor in breast cancer. Proc. Natl. Acad. Sci. USA.

[11] Jiang F.N., Dai L.J., Yang S.B., Wu Y.D., Liang Y.X., Yin X.L., Zou C.Y. (2020). Increasing of FKBP9 can predict poor prognosis in patients with prostate cancer. Pathol. Res. Pract..

[12] Xu H., Liu P., Yan Y., Fang K., Liang D., Hou X., Zhang X., Wu S., Ma J., Wang R. (2020). FKBP9 promotes the malignant behavior of glioblastoma cells and confers resistance to endoplasmic reticulum stress inducers. J. Exp. Clin. Cancer Res..

[13] Ge Y., Xu A., Zhang M., Xiong H., Fang L., Zhang X., Liu C., Wu S. (2017). FK506 Binding Protein 10 Is Overexpressed and Promotes Renal Cell Carcinoma. Urol. Int..

[14] Cai H.Q., Zhang M.J., Cheng Z.J., Yu J., Yuan Q., Zhang J., Cai Y., Yang L.Y., Zhang Y., Hao J.J. (2021). FKBP10 promotes proliferation of glioma cells via activating AKT-CREB-PCNA axis. J. Biomed. Sci..

[15] Wang R.G., Zhang D., Zhao C.H., Wang Q.L., Qu H., He Q.S. (2020). FKBP10 functioned as a cancer-promoting factor mediates cell proliferation, invasion, and migration via regulating PI3K signaling pathway in stomach adenocarcinoma. Kaohsiung J. Med. Sci..

[16] Liang L., Zhao K., Zhu J.H., Chen G., Qin X.G., Chen J.Q. (2019). Comprehensive evaluation of FKBP10 expression and its prognostic potential in gastric cancer. Oncol. Rep..

[17] Ramadori G., Ioris R.M., Villanyi Z., Firnkes R., Panasenko O.O., Allen G., Konstantinidou G., Aras E., Brenachot X., Biscotti T. (2020). FKBP10 Regulates Protein Translation to Sustain Lung Cancer Growth. Cell Rep..

[18] Zhao J., Wang Y., Gao J., Wang Y., Zhong X., Wu X., Li H. (2021). A nine-gene signature to improve prognosis prediction of colon carcinoma. Cell Cycle.

[19] Zhang Z., Huang L., Li J., Wang P. (2022). Bioinformatics analysis reveals immune prognostic markers for overall survival of colorectal cancer patients: A novel machine learning survival predictive system. BMC Bioinform..

[20] Chen Z., He L., Zhao L., Zhang G., Wang Z., Zhu P., Liu B. (2022). circREEP3 Drives Colorectal Cancer Progression via Activation of FKBP10 Transcription and Restriction of Antitumor Immunity. Adv. Sci..

[21] Quinn M.C., Wojnarowicz P.M., Pickett A., Provencher D.M., Mes-Masson A.M., Davis E.C., Tonin P.N. (2013). FKBP10/FKBP65 expression in high-grade ovarian serous carcinoma and its association with patient outcome. Int. J. Oncol..

[22] Alqudah A., AbuDalo R., Qnais E., Wedyan M., Oqal M., McClements L. (2022). The emerging importance of immunophilins in fibrosis development. Mol. Cell Biochem..

[23] Petrosyan A. (2019). Unlocking Golgi: Why Does Morphology Matter?. Biochemistry.

[24] Coleman O.I., Haller D. (2019). ER Stress and the UPR in Shaping Intestinal Tissue Homeostasis and Immunity. Front. Immunol..

[25] Song M., Li Y., Miao M., Zhang F., Yuan H., Cao F., Chang W., Shi H., Song C. (2020). High stromal nicotinamide N-methyltransferase (NNMT) indicates poor prognosis in colorectal cancer. Cancer Med..

