# Supplementary material for: Subcellular Expression Patterns of FKBP Prolyl Isomerase 10 (FKBP10) in Colorectal Cancer and Its Clinical Significance
Source: Int J Mol Sci. 2023 Jul 13;24(14):11415. doi: 10.3390/ijms241411415 (PMC10380463; doi:10.3390/ijms241411415)
Supplement: Supplementary file 1 [file ijms-24-11415-s001.zip › ijms-2478103-supplementary.pdf]

International Journal of *Molecular Sciences*

MDPI

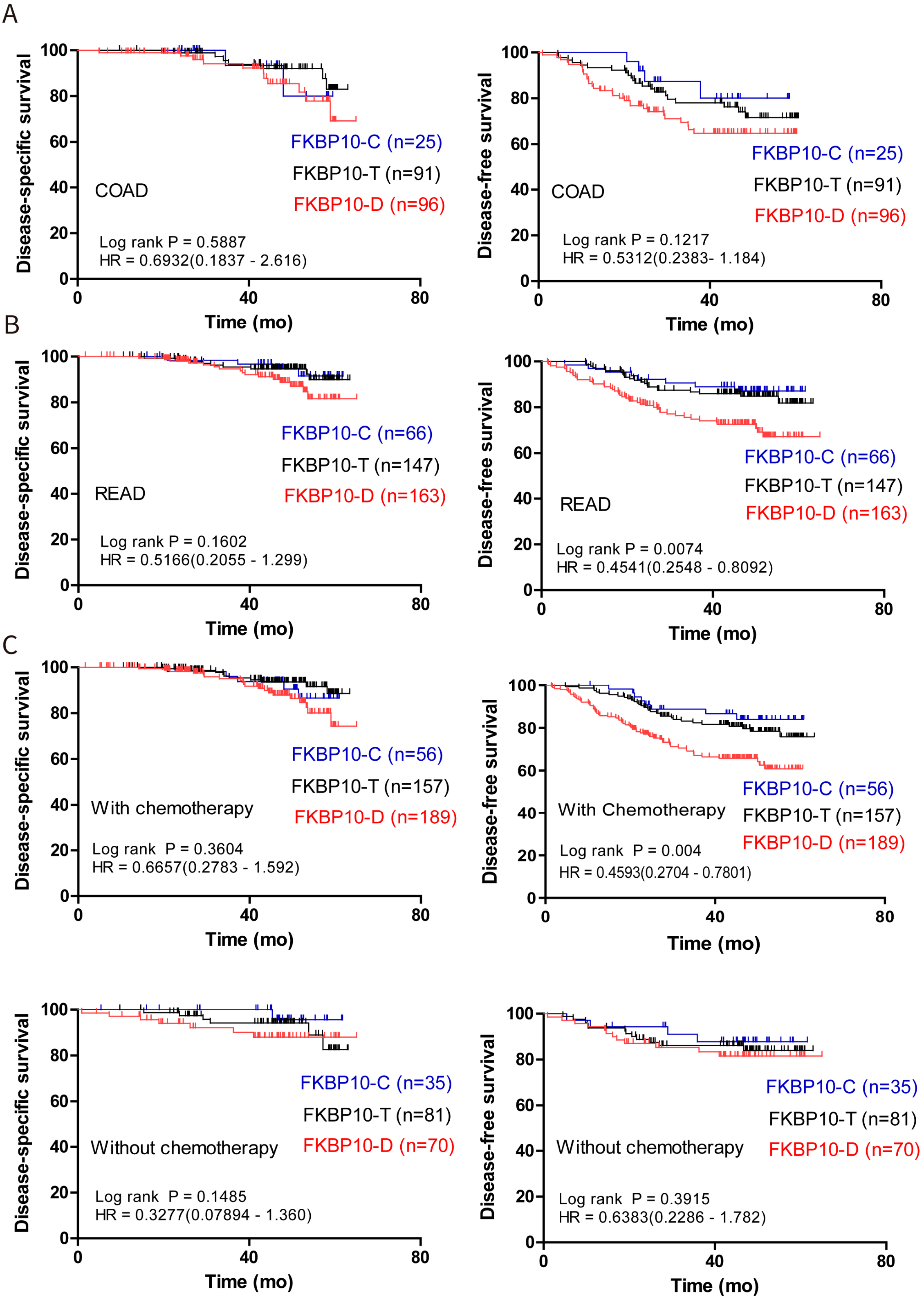

Figure S1. The associations between the subcellular expression patterns of FKBP10 and patients' outcome considering the tumor location and chemotherapy employment. Kaplan-Meier analysis of patients with (A) colorectal carcinoma, (B) rectal carcinoma, or (C) chemotherapy employment. Log-rank P values were representative of three subgroups and hazard ratios (HRs) were representative of FKPB10-C vs. FKPB10-D from Kaplan-Meier analysis with log-rank test. COAD, colorectal carcinoma; READ, rectal carcinoma.

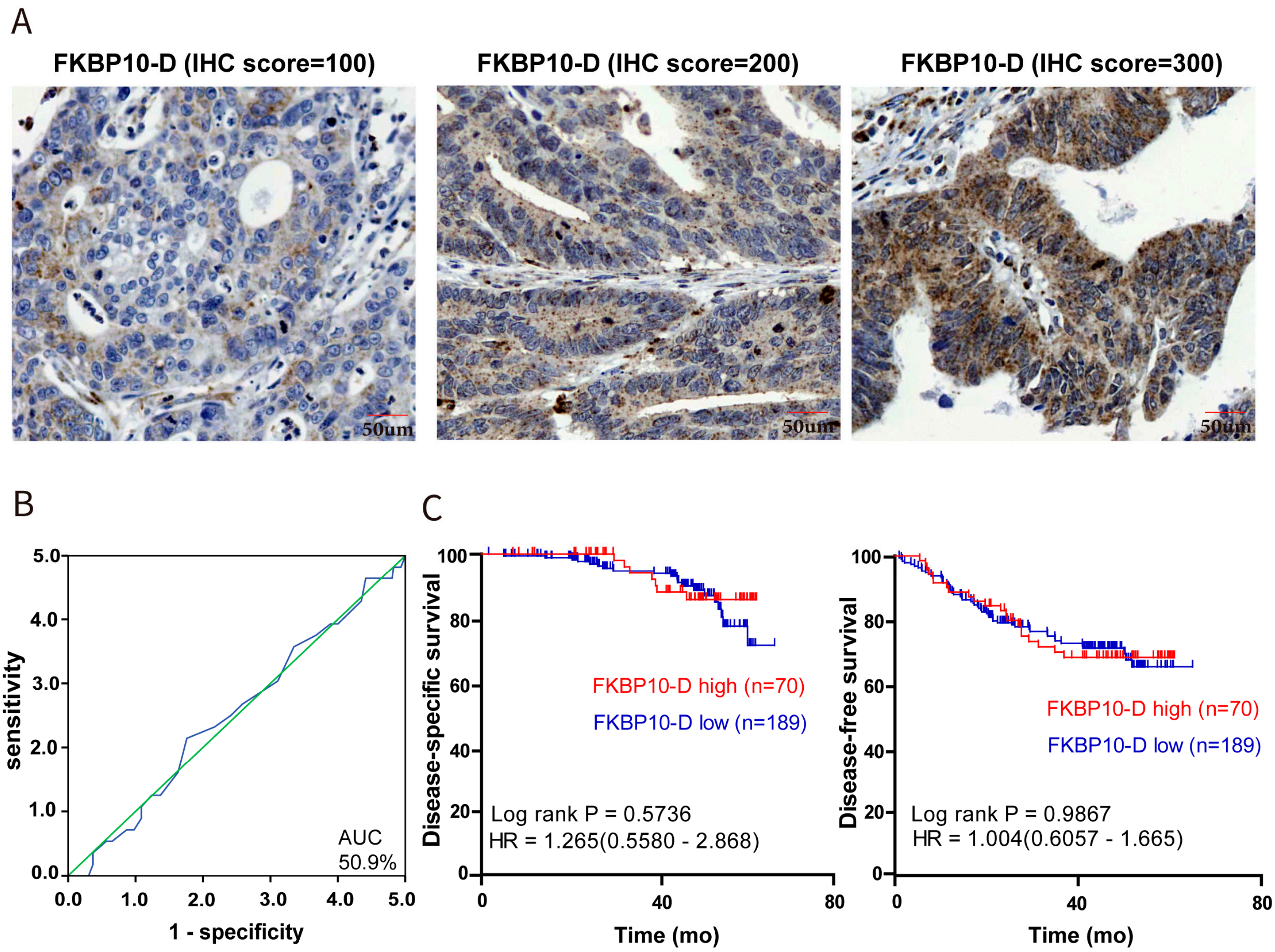

Figure S2. High epithelial FKBP10-D expression was not prognostic in CRC. (A) Representative images of immunostaining of the FKBP10-D in cancer tissues, respectively. (B) ROC curves analysis of epithelial FKBP10-D expression and patients' outcome in the CRC cohort. (C) The associations of epithelial FKBP10-D expression with the overall DSS and DFS of CRC patients.
